# Supercritical CO_2_ extraction of naringenin from Mexican oregano (*Lippia graveolens*): its antioxidant capacity under simulated gastrointestinal digestion

**DOI:** 10.1038/s41598-023-50997-2

**Published:** 2024-01-11

**Authors:** Manuel Adrian Picos-Salas, Nayely Leyva-López, Pedro de Jesús Bastidas-Bastidas, Marilena Antunes-Ricardo, Luis Angel Cabanillas-Bojórquez, Miguel Angel Angulo-Escalante, J. Basilio Heredia, Erick Paul Gutiérrez-Grijalva

**Affiliations:** 1 Centro de Investigación en Alimentación y Desarrollo A.C., Carretera a Eldorado Km 5.5, Col. Campo El Diez, 80110 Culiacán, Sinaloa México; 2 Posdoc CONAHCYT-Centro de Investigación en Alimentación y Desarrollo A.C., Carretera a Eldorado Km 5.5, Col. Campo El Diez, 80110 Culiacán, Sinaloa México; 3 Tecnologico de Monterrey, Centro de Biotecnología FEMSA, Escuela de Ingeniería y Ciencias, Av. Eugenio Garza Sada 2501 Sur, Monterrey, NL 64849 México; 4 Tecnologico de Monterrey, Institute for Obesity Research, Av. Eugenio Garza Sada 2501 Sur, 64849 Monterrey, NL México; 5 Functional Foods and Nutraceuticals Laboratory, Cátedras CONAHCYT-Centro de Investigación en Alimentación y Desarrollo A.C., Carretera a Eldorado Km 5.5, Col. Campo El Diez, 80110 Culiacán, Sinaloa México

**Keywords:** Natural products, Green chemistry

## Abstract

A supercritical CO_2_ method was optimized to recover naringenin-rich extract from Mexican oregano (*Lippia graveolens*), a flavanone with high antioxidant and anti-inflammatory activity. The effect of the extraction parameters like pressure, temperature, and co-solvent on naringenin concentration was evaluated. We used response surface methodology to optimize the naringenin extraction from oregano; the chemical composition by UPLC-MS of the optimized extract and the effect of simulated gastrointestinal digestion on its antioxidant capacity and total phenolic content were also evaluated. The optimum conditions were 58.4 °C and 12.46% co-solvent (ethanol), with a pressure of 166 bar, obtaining a naringenin content of 46.59 mg/g extract. Also, supercritical optimized extracts yielded high quantities of cirsimaritin, quercetin, phloridzin, apigenin, and luteolin. The results indicated that the naringenin-rich extract obtained at optimized conditions had higher total phenolic content, antioxidant capacity by TEAC and ORAC, and flavonoid content, compared with the methanolic extract, and the simulated gastrointestinal digestion reduced all these values.

## Introduction

Oregano is a group of plants with common attributes such as aroma and flavor, *Lippia graveolens*, also known as Mexican oregano, is one the most widely distributed species. This plant has a variety of lipophilic compounds in its essential oil, and most studies are related to them^1^; however, the content of phenolic compounds has been of interest in recent studies^2,3^. Phenolic compounds are secondary metabolites ubiquitous in the plant kingdom; these molecules act as a defense mechanism against biotic and abiotic stress. Among them, flavonoids are some of the most important compounds in this category; they are synthesized by the phenylpropanoid pathway, forming a basic structure of two phenyl rings (A and B) united by a pyran ring (C)^4^. These compounds have been attributed with health benefits; for instance, quercetin has been shown to induce apoptosis in pancreatic cancer cell lines^5^, and cirsimaritin exhibited therapeutic effects in damaged beta cells^6^. Furthermore, naringenin (4ʹ,5,7-trihydroxyflavanone) has antioxidant, anti-inflammatory^7^, and anti-proliferative^8^ properties. Also, although commonly found in citric fruits, several studies indicate that naringenin is one of the most abundant flavonoids in *Lippia graveolens*^2,3,9^.

Commonly, flavonoids are extracted from plant matrixes using organic solvents, which can harm the environment and human health to various degrees; for example, the FDA classified solvents like methanol as class 2, with limited use because of its toxicity^10^. In this regard, alternative extraction methods have been employed to extract phenolic compounds, mitigating these drawbacks; microwave-assisted extraction, ultrasound-assisted extraction, pressurized liquid extraction, and supercritical CO_2_ extraction are the most common ones^11,12^. In this sense, supercritical CO_2_ extraction is an effective method to extract compounds of diverse polarity based on the behavior of CO_2_ in supercritical state (solvating like a liquid and diffusing like a gas). In this state, the CO_2_ can enter and disrupt the plant cell and extract desired compounds according to the process conditions; however, this solvent is non-polar, and adding a polar cosolvent allows the extraction of compounds of this nature^13^. The ability to tune parameters of the process like temperature, pressure, and cosolvent proportion allows this technology to have better characteristics compared to other techniques, including higher selectivity and yields of the desired compound, as well as short extraction times; furthermore, the supercritical conditions of CO_2_ are relatively low (31 °C and 74 bar), avoiding thermal degradation of phytochemicals and reducing power consumption^14,15^. Regarding extraction of phenolic compounds from plant matrixes, studies have determined conditions of 36–60 °C, pressure between 100 and 300 bar, and ethanol (cosolvent) proportion of up to 20% had been effective to obtain high quantities of these compounds^3,16–18^.

Furthermore, phenolic compounds must be bioaccessible and bioavailable to exert their bioactivities; in this context, bioaccessibility is defined as the proportion of compounds released from the food matrix and available for intestinal absorption^19^. In general, the gastrointestinal process induces changes in the structure of phenolic compounds by the changes in pH and the presence of digestive enzymes, affecting its bioaccessibility^20,21^.

Thus, this research aimed to optimize a supercritical CO_2_ process to obtain a naringenin-rich extract from Mexican oregano and evaluate the effect of a simulated gastrointestinal digestion on its antioxidant capacity and flavonoid content.

## Results and discussion

### Response surface methodology analysis and optimization

The model adjusted for the linear terms of temperature and cosolvent, and the quadratic term cosolvent^2^, for the response (naringenin content), without a lack of fit and a coefficient of determination (R^2^) of 0.8079 (Table 1); indicating that the adjusted terms explain 80.79% of the variability of the response. The prediction for naringenin content is shown in Eq. (1) for the codified values and in Eq. (2) for natural values.

**Table 1:** Analysis of variance for naringenin content of *Lippia graveolens* supercritical CO_2_ extraction using response surface quadratic model.

Source	Degrees of freedom	Adjusted sum of squares	Adjusted mean squares	F value	p value
Model	4	1,172,976,154	293,244,039	15.77	0.000
Pressure	1	6,658,162	6,658,162	0.36	0.559
Temperature	1	169,721,621	169,721,621	9.13	0.009
Cosolvent	1	471,102,758	471,102,758	25.34	0.000
Cosolvent^2^	1	525,493,613	525,493,613	28.26	0.000
Error	15	278,911,464	18,594,098		
Lack of fit	10	240,295,359	24,029,536	3.11	0.111
Pure error	5	38,616,105	7,723,221		
Total	19	1,451,887,618			
R^2^ = 80.79%					
Adjusted R^2^ = 75.67%					
CV = 12.70%					

1 $${\text{Naringenin}}\;{\text{content}} = 38051.08 + 698.24\;{\text{X}}_{1} + 3525.28\;{\text{X}}_{2} + 5873.30\;X_{3} - 5984.14\;{\text{X}}_{3}^{2}$$

2 $${\text{Naringenin}}\;{\text{content}} = - 20953 + 14\;{\text{P}} + 441\;{\text{T}} + 5962\;{\text{C}} - 239.4\;{\text{C}}^{2}$$

**Figure 1:**
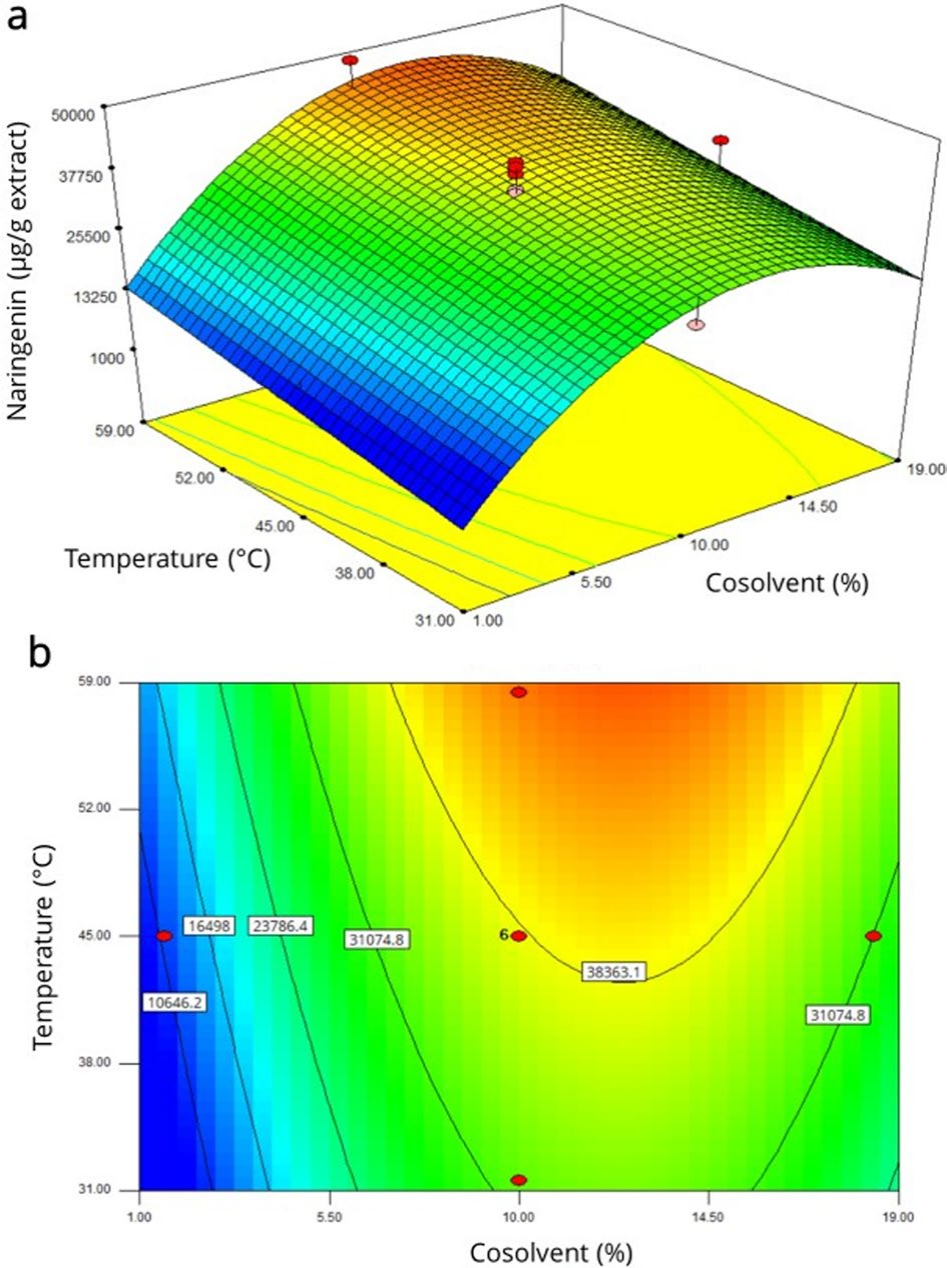
Response surface (**a**) and contour plot (**b**) for naringenin content.

The contour plot and response surface indicate a superior naringenin content at high temperatures and less than 15% of cosolvent, while pressure did not affect the extraction of this flavanone (Fig. 1).

The desirability optimization showed a predicted value of 46,590 µg/g extract at the optimum conditions of 58.4 °C (temperature) and 12.46% (cosolvent), with a pressure of 166 bar, showing a desirability value of 0.9384, being a value of 1 the optimal performance of the factors^22^.

After replicating the optimum conditions four times, the naringenin content was 51,457.79 ± 5831.36 µg/g extract in the supercritical extract at optimum condition (SE), indicating the model accurately predicted the response. Supercritical CO_2_ extraction is a highly sensitive method to process conditions, including (but not limited to) temperature, pressure, and cosolvent proportion. In this sense, our optimization allowed the extraction of high quantities of naringenin, related to the moderate temperature (less than 60 °C), suggesting that an increase in vapor pressure by increasing temperature affects the solubility of this flavanone in supercritical CO_2_ more than the CO_2_ density, which decreases while increasing temperature^23^. Moreover, low pressure is desired to improve selectivity, as well as reduce the extraction of oils, waxes, and other no desriables compounds^24^, and enhance flavonoid extraction^25^. In addition, naringenin solubility was enhanced by the addition of ethanol as cosolvent due to the polarity of the flavonoid; similarly, as seen in other plant matrixes, the use of this alcohol as cosolvents is effective in extracting this compound^17,26^; however, increasing the cosolvent proportion more than 12.46% reduced the naringenin extraction, attributed to a decrease in supercritical CO_2_ selectivity^14^, likewise, high quantities of cosolvent also decrease the influence of temperature in the extraction^27^; also, this effect can also be explained as an excess of cosolvent increase the interaction between it and the solute, reducing supercritical fluid-solute interaction^28^, and prevents supercritical CO_2_ from diffusing through the plant matrix^29^.

To the best of our knowledge, there are not studies about the specific extraction and optimization of naringenin from plants using supercritical CO_2_; although, it had been extracted from matrixes like spearmint (*Mentha spicata* L.) and *Strobilanthes crispus* leaves at similar process conditions (60 °C, 200 bar, 60 min; and 60 °C, 100 bar, 80 min; respectively)^16,30^.

### Flavonoid quantification by UPLC-MS

The content of flavonoids of the undigested and digested extracts is shown in Table 2, and their chromatograms in Supplementary Fig. S1. In the SE, the most abundant flavonoids were naringenin, cirsimaritin, and quercetin; while in the methanolic extract (ME) the major flavonoids were naringenin, phloridzin, and cirsimaritin. Furthermore, the flavonoid content of each flavonoid was higher in the SE, except for kaempferol, luteolin, and phloridzin. Interestingly, after the simulated digestion, the flavonoid content decreased significantly; in addition, most of the flavonoids only showed a bioaccessibility of 1% or lower, and only apigenin of the intestinal phase of the supercritical extract (ISE) and phloridzin of the intestinal phase of the methanolic extract (IME) showed a bioaccessibility of 4.83% and 7.09%, respectively; in this sense, the presence of a sugar substituent in the structure of phloridzin confers a more stable structure compared with the other aglycones^31^. Also, apigenin has better solubility at more alkaline pH (Supplementary Fig. S2), that allows better bioaccessibility at the intestinal phase, due to having a pKa of 7.02 at C5 of the A ring, with a deprotonated hydroxyl group and being more soluble. Compared with other flavonoids, naringenin (pKa = 7.86, at C7), cirsimaritin (pKa = 7.87, at C5), and quercetin (pKa = 7.58 at C7) are mostly in the protonated form at pH 7, reducing their solubility and, in consequence, their bioaccessibility in the intestinal phase.

**Table 2:** Identification and quantification of flavonoids by UPLC-MS in the supercritical extract (SE), methanolic extract (ME), intestinal phase of supercritical extract (ISE), and intestinal phase of methanolic extract (IME).

RT (min)	Parent m/z	Daughter m/z	Flavonoid	Content (µg/g extract)				Bioaccessibility (%)	
				SE	ISE	ME	IME	ISE	IME
3.41	435.00	167.00* 273.00 297.00	Phloridzin	856.36 ± 61.55^b^	NQ	2909.60 ± 7.06^a^	206.40 ± 19.19^c^	0	7.09
3.80	301.10	107.19 151.14 179.16*	Quercetin	3410.98 ± 285.34^a^	0.84 ± 0.03^c^	1476.08 ± 72.55^b^	0.25 ± 0.01^c^	0.02	0.02
3.80	285.00	68.90 153.00*	Luteolin	253.31 ± 3.98^a^	3.37 ± 0.27^c^	370.98 ± 64.25^a^	0.69 ± 0.03^c^	1.30	0.19
4.09	269.00	90.90 153.00*	Apigenin	528.78 ± 30.40^a^	25.56 ± 0.64^c^	246 ± 24.89^b^	1.10 ± 0.11^c^	4.83	0.45
4.17	273.10	119.09 147.11 153.09*	Naringenin	51,457.79 ± 2915.68^a^	463.57 ± 22.87^c^	9850.09 ± 584.69^b^	128.50 ± 11.13^c^	0.90	1.30
4.54	315.03	136.06 254.16 282.15*	Cirsimaritin	4729.49 ± 254.55^a^	47.68 ± 4.31^c^	1842.19 ± 30.72^b^	1.49 ± 0.15^c^	1.01	0.08

Data represent mean ± SEM (n = 4 for SE and ISE, n = 3 for ME and IME). Ions with asterisk (*) were used to quantification, while ions without it were used for confirmation. Different letters in the same row indicate significant difference by Tukey’s Test (p < 0.05).

*RT* retention time, *NQ* not quantified.

The differences in flavonoids of both extracts are attributed to the different conditions of the processes; the supercritical extraction was carried out in conditions of polarity, solvent density, and viscosity adequate to extract higher quantities of naringenin and, at the same time, these conditions were adequate to the other flavonoids. Besides, the XLogP3-AA of the flavonoids in higher quantities (naringenin and cirsimaritin) is are similar, 2.4 and 2, respectively; compared with the rest of the compounds (ranged from 1.2 to 1.7); meaning the process conditions where adequate to extract compounds of these polarities. The presence of naringenin as the most abundant compound is in concordance with previous studies^3^, and other flavonoids have also been reported^32^.

Also, the low stability of the flavonoids during the simulated digestion can be attributed to the pH changes during the digestion steps. In general, flavonoids are more stable at gastric pH conditions, compared to the neutral pH of the intestine, favoring autoxidation reaction^33^. In addition, possible interactions between the compounds with the digestive enzymes could reduce their bioaccessibility^34,35^, as phenolic compounds precipitate proteins by binding to them^36,37^.

Our study is in concordance with other where naringenin, cirismaritin are ones of the major flavonoids^2,9^; also, a study of Bernal-Millán et al.^32^, showed that the different extraction methods did not affect the naringenin content in their extracts. In contrast, our study achieved the desired conditions to enhance the naringenin extraction.

### Total phenolic content

The extraction method significantly affected the total phenolic content, as shown in Table 3, where the SE had a higher value than the ME (p < 0.05). This difference can be attributed to the better selectivity of the supercritical CO_2_, a process where the control of the variables enhances the phenolic compound extraction yield^38^, and this has also been reported for leaves and berries of myrtle^39^. The supercritical extraction process allowed to extract phenolic compounds in high quantities compared to the methanolic extraction, including naringenin; however, our results are lower compared to a previous study by Picos-Salas, et al.^3^, where the TPC was 354 mg GAE/g of extract; as well against the methanolic extract from Martínez-Rocha, et al.^40^, with a TPC of 270.25 mg GAE/g extract; which can be attributed to the plant growth conditions. In addition, the process in the present work is focused on enhancing the extraction of naringenin, probably avoiding the extraction of other compounds that can react to the Folin-Ciocalteu reagent; in addition, changes in extraction parameter are reflected in the total phenolic content value^41^.

**Table 3:** Total phenolic content and antioxidant capacity of the supercritical extract (SE), methanolic extract (ME), intestinal phase of supercritical extract (ISE), and intestinal phase of methanolic extract (IME).

Extract	TPC (mg GAE/g extract)	TEAC (mmol TE/g extract)	ORAC (µmol TE/g extract)
SE	150.79 ± 3.44^a^	2327.60 ± 71.52^a^	6923.65 ± 57.20^a^
ISE	21.81 ± 0.98^c^	116.44 ± 4.37^b^	129.52 ± 8.89^c^
ME	91.80 ± 4.68^b^	2259.99 ± 169.81^a^	3577.08 ± 195.09^b^
IME	19.44 ± 0.44^c^	112.51 ± 4.33^b^	106.56 ± 4.32^c^

Data represent mean ± SEM (n = 4 for SE and ISE, n = 3 for ME and IME). Different letters in the same column indicate significant difference by Tukey’s Test (p < 0.05).

### Antioxidant capacity

There was no significant difference between the SE and ME TEAC values (Table 3). On the other hand, the SE showed significantly higher ORAC values than the ME (Table 3). The simulated digestion process caused a significant loss in antioxidant capacity; the TEAC values decreased to 5% for both extraction methods, while the ORAC results decreased to 1.87% for the ISE and 2.98% for the IME; this compared with the extract without simulated digestion.

It has been established that phenolic compounds suffer degradation during gastrointestinal digestion caused by the pH changes and presence of enzymes; in this sense, the simulated digestion provided harsh conditions to the compounds, causing changes in their structures by deprotonating their –OH radicals, thus, reducing their antioxidant capacity^42,43^.

The TEAC assay is based in a mixed mode, where electron transfer (ET), hydrogen atom transfer (HAT), and proton-coupled electron transfer mechanisms happen at different proportions depending on the solvent and pH of the reaction^44^; however, phenolic compounds respond better to assays related to HAT mechanism; this caused no significant differences between the antioxidant capacity of both undigested extracts. On the contrary, the ORAC assay is based on the HAT mechanism, which is predominant in phenolic compounds like flavonoids^45^.

These results indicate that the higher content of naringenin and other flavonoids, like cirsimaritin and quercetin, contributed to the better antioxidant capacity of the SE. Even if naringenin is the most abundant flavonoid in the extracts, its antioxidant power is rather low compared to other flavonoids due to the absence of a catechol group in the B ring and lack of C2–C3 double bond^46^; however, the relatively high presence of naringenin and its synergy with quercetin (with a catechol group in B ring) and cirsimaritin could influence the higher antioxidant values, and confer better antioxidant power to the extract. In this sense, the mixture of naringenin and quercetin has shown better antioxidant activity against the sum of their individual components^47^; also, the synergism phenomenon has been reported in leaves of crabapples, as the individual flavonoids showed lower values compared to mixtures of them^48^.

Compared to a previous study in our research group, the ORAC values were 1.4 times higher, which can be attributed to our specific process of extracting naringenin in high quantities. In contrast, in our previous work, the process was not optimized to a specific compound^3^. Also, compared to other supercritical extracts, our results were higher than both dry and fermented orange pomace extract (370–1260 µmol TE/g extract)^49^; as well against the peel oil extract from mandarin (*Clementina orogrande*) (433.46–738.68 µmol TE/g oil)^50^. In both cases, ethanol was used as cosolvent, enhancing the antioxidant response by increasing the flavanones like naringenin, naringin, and hesperetin. Also, previous studies in our research group showed lower TEAC values compared to current findings, this is due to less specific extraction condition, allowing of the extraction of a variety of compounds that can react to the ABTS radical^3^; but compared to other supercritical extracts rich in phenolic compounds, like from *Sida rhombifolia* leaves^51^ and cacado pod husk^52^, our results were higher.

## Conclusion

The optimized conditions to obtain naringenin from oregano using supercritical CO_2_ were low pressure (166 bar) and cosolvent proportion (12.46%), and moderate temperature (58.4 °C). In addition, the optimized process yielded more cirsimaritin, quercetin, and apigenin, compared with a methanolic extraction. However, the bioaccessibility of these compounds was low, as seen in the reduction of their content and the antioxidant capacity of the extract. The use of supercritical CO_2_ enhances the extraction of naringenin from oregano (*Lippia graveolens*), and it was an effective method to obtain this flavanone; therefore, the naringenin-rich extract with health benefits can be used in different industries, and we suggest incorporating encapsulation techniques to increase the bioaccessibility of these extracts.

## Materials and methods

### Sample preparation

Wild *Lippia graveolens* were obtained in Santa Gertrudis, Durango, México (coordinates: N 23° 32′ 43.8″ W 104° 22′ 20.8″). The aerial parts (flowers, leaves, and small stems) were dried using an Excalibur Food Dehydrator Parallax Hyperware (Sacramento, CA) at 40 °C for 24 h. Dried samples were grounded to a fine powder using an Ika Werke M20 mill grinder (IKA, Germany) and were stored at − 20 °C for further experiments.

### Reagents and chemicals

The nitrogen and CO_2_ were purchased from Linde (Culiacán, Sinaloa, Mexico), methanol and ethanol were obtained from Fermont (Mexico). The rest of the reagents were purchased from Sigma-Aldrich (St. Louis, MO, USA).

### Conventional extraction

The conventional extraction was performed according to a previous report by Picos-Salas et al.^3^ with modifications. In brief, 0.1 g of oregano powder was mixed with 10 mL of methanol (100%) in constant shaking for 2 h. Afterward, the mixture was centrifugated at 11,627 g for 15 min at 4 °C using a Z 36 HK centrifuge (HERMLE, Germany). Then, the supernatant was collected and dried using a rotary evaporator R-300 (Buchi, Switzerland), followed by drying with nitrogen to ensure complete solvent remotion, and stored at − 20 °C until further analysis. Extraction was carried by triplicate (n = 3).

### Supercritical CO_2_ extraction

Naringenin-rich extracts were obtained from *Lippia graveolens* using a MV-10 ASFE extractor (Waters Corporation, MA, USA), using CO_2_ as solvent and ethanol (99.9%) as cosolvent. The conditions used were selected based on literature research and preliminary studies. In brief, 2.5 g of sample was placed in a 10 mL vessel; the extraction was carried at the pressure, temperature, and cosolvent proportion according to the central composite design (Table 4). All extractions were carried at the same CO_2_ + cosolvent flow (5 mL/min), with static and dynamic extraction times (30 and 45 min each). All extracts were dried in a vacuum concentrator Multivapor P-12 (Buchi, Switzerland), resuspended in ethanol (99.9%), and stored at − 20 °C for further studies.

**Table 4:** Coded and natural variables and response results for the central composite design.

Standard order	Pressure (X_1_, bar)	Temperature (X_2_, °C)	Cosolvent (X_3,_ %)	Naringenin (µg/g extract)
1	200 (− 1)	37 (− 1)	5 (− 1)	27,849.33
2	300 (1)	37 (− 1)	5 (− 1)	27,733.86
3	200 (− 1)	53 (1)	5 (− 1)	30,811.21
4	300 (1)	53 (1)	5 (− 1)	24,591.80
5	200 (− 1)	37 (− 1)	15 (1)	31,677.42
6	300 (1)	37 (− 1)	15 (1)	34,237.62
7	200 (− 1)	53 (1)	15 (1)	34,772.38
8	300 (1)	53 (1)	15 (1)	39,104.50
9	165.9 (− 1.682)	45 (0)	10 (0)	32,744.89
10	334.1 (1.682)	45 (0)	10 (0)	40,032.23
11	250 (0)	31.5 (− 1.682)	10 (0)	26,638.12
12	250 (0)	58.4 (1.682)	10 (0)	49,163.31
13	250 (0)	45 (0)	1.6 (− 1.682)	7460.86
14	250 (0)	45 (0)	18.4 (1.682)	36,552.03
15	250 (0)	45 (0)	10 (0)	36,074.36
16	250 (0)	45 (0)	10 (0)	40,344.00
17	250 (0)	45 (0)	10 (0)	37,395.23
18	250 (0)	45 (0)	10 (0)	36,611.09
19	250 (0)	45 (0)	10 (0)	43,603.19
20	250 (0)	45 (0)	10 (0)	41,637.04

### Simulated digestion

An in vitro digestion model was carried out following the protocol by Brodkorb et al.^53^, which consisted in a 3-step process that simulates oral, gastric, and intestinal digestion (see Supplementary Table S1). In brief, in a 50 mL tube, 1 mL of extract was mixed with 0.8 mL of oral solution, 5 µL of 0.3 M CaCl_2_, 0.1 mL of amylase (75 U/mL), 0.095 mL of distilled water, and incubated in an oscillator for 2 min at 37 °C. Afterward, the solution was mixed with 1.6 mL of gastric solution, and pH was adjusted to 3; then, 1 µL of 0.3 M CaCl_2_, 0.1 mL of pepsin (2000 U/mL), and 0.1 mL of lipase (60 U/mL) were added, and pH adjusted to 3; next, 0.199 mL of distilled water was added, and the solution was incubated during 2 h at 37 °C. Finally, 2.2 mL of gastric solution was added to the mix, and pH was adjusted to 7; then, 8 µL of 0.3 M CaCl_2_ and 1 mL of pancreatin (100 U/mL) were added, and pH was adjusted to 7; later, 0.792 mL of distilled water were added, and the solution was incubated for 2 h at 37 °C. After the simulated digestion process, methanol (100%) was added to precipitate proteins in 1:1 v/v, and the solution was placed at − 20 °C for 20 min; afterward, it was centrifugated at 11,627 g for 10 min and 4 °C. Finally, the supernatant was collected and named as supercritical extract intestinal phase (ISE) or methanolic extract intestinal phase (IME). Bioaccessibility (%) of each flavonoid was calculated as seen in Eq. (3):

3 $$Bioaccessibility\left( \% \right) = \frac{{Flavonoid\;in\;intestinal\;phase}}{{Flavonoid\;in\;crude\;extract}} \times 100$$

where both flavonoid in intestinal phase and crude extract are expressed in µg of flavonoid/g extract.

### Flavonoid content by UPLC-MS

The flavonoids in the extracts were evaluated based on the methodology by Bernal-Millán et al.^54^ using a UPLC class H (Waters Corporation, USA) coupled to a G2-XS QT mass analyzer (Quadrupole and Time of Flight). Flavonoids were separated using a UPLC BEH C18 column (1.7 μm × 2.1 mm × 100 mm) at 40 °C. Gradient elution was conducted with water-formic acid 0.1% (A) and acetonitrile (B) at the flow rate of 0.3 mL/min. The following gradient was used: 0 min, 95% (A); 5 min, 70% (A); 9 min, 30% (A); 14 min, 0% (A); 14.5 min, 0% (A); 15 min, 95% (A); and 16 min, 95% (A). Electrospray (ESI) was used for compound ionization, and the mass analysis condition was: capillary voltage of 1.5 kV, sampling cone of 30 V, desolvation gas of 800 L/h, and a temperature of 500 °C. A 0–30 V collision ramp was used. The flavonoids were identified and quantified by comparing them with a calibration curve using the corresponding standards (Supplementary Fig. S3).

### Total phenolic content

The total phenolic content (TPC) was done as reported by Swain and Hillis^55^ with modifications. In brief, 10 µL of the extract was mixed with 230 µL of distilled water and 10 µL of the Folin–Ciocalteu reagent in a 96-well microplate. Afterward, 25 µL of 4 N Na_2_CO_3_ was added, and the mixture was incubated in darkness for 2 h. Then, absorbance was measured at 725 nm using a Synergy HT microplate reader (Bio-Tek Instruments, Inc., VT, USA). Results were expressed as mg of gallic acid equivalents per g of extract (mg GAE/g extract).

### Oxygen radical absorbance capacity (ORAC)

The antioxidant capacity was measured by the ORAC assay described by Huang et al.^56^ using fluorescein as a fluorescent probe and 2,2′-azobis(2-methylpropionamidine) dihydrochloride (AAPH) as a peroxyl radical. A reaction mixture containing 25 µL of extract, 75 µL of 95.8 µM AAPH, and 200 µL of 0.96 µM fluorescein was put in a black-walled, clear-bottom 96-well microplate, and the reaction started when the AAPH was added. Phosphate buffer was used as a blank. The loss of fluorescence was measured every 70 s for 70 min at 485 nm for excitation and 580 nm for emission using a microplate reader. Results were expressed as µmol of Trolox equivalents per g of extract (µmol TE/g extract).

### Trolox equivalent antioxidant capacity (TEAC)

The TEAC assay was carried out according to Thaipong et al.^57^ with some modifications. For this, the ABTS^·+^ radical was formed by mixing 7.4 mM ABTS with 2.4 mM potassium persulphate at a 1:1 ratio and incubated at room temperature in darkness during 12–16 h before use. Afterward, the radical was diluted with ethanol (99.9%) until an absorbance of 0.70 ± 0.02 at 734 nm. The reaction started after adding 10 µL of the extract with 190 µL of the ABTS^·+^ in a 96-well microplate, followed by incubation during 2 in darkness and absorbance measuring at 734 nm in a microplate reader. The ABTS^·+^ radical was used as a blank. Results were expressed as mmol of Trolox equivalents per g of extract (mmol TE/g extract).

### XLogP3-AA and logD data obtention

XlogP3-AA values were obtained using PubChem database^58^, and the LogD values using the online platform Chemicalize^59^.

### Experimental design

The naringenin supercritical CO_2_ extraction was optimized by the response surface methodology using the desirability function. A central composite design with three factors was used, namely pressure (X_1_, bar), temperature (X_2_, °C), and cosolvent proportion (X_3_, %) (Table 4), using the software Design-Expert 7.0 (Stat-Ease Inc., MN, USA). The naringenin content was used as a response variable and was measured by UPLC-MS using a naringenin standard as specified before. The optimum conditions were carried out in quadruplicate (n = 4).

Flavonoid quantification by UPLC-MS, TPC, ORAC, and TEAC were analyzed by an analysis of variance (ANOVA) with two factors (supercritical and methanolic extract) and two levels each one (extract without digestion and intestinal phase), and each analysis was performed by triplicate for the methanolic extracts, and by quadruplicate for the supercritical extract at optimized condition; also, mean comparisons were evaluated by Tukey’s HSD test using the software Minitab 19 (Minitab LLC, PA, USA). A level of p < 0.05 was considered a significant difference. Data were reported as mean ± standard error of the mean (SEM).

### Supplementary Information

Supplementary Figure S1.

Supplementary Figure S2.

Supplementary Figure S3.

Supplementary Table S1.

## Data Availability

All data generated or analyzed during this study is included in article and its supplementary information files.

## References

[1] Leyva-López N, Gutiérrez-Grijalva E, Vazquez-Olivo G, Heredia J (2017). Essential oils of oregano: Biological activity beyond their antimicrobial properties. Molecules.

[2] Arias J (2020). Optimization of flavonoids extraction from *Lippia graveolens* and *Lippia origanoides* chemotypes with ethanol-modified supercritical CO_2_ after steam distillation. Ind. Crop. Prod..

[3] Picos-Salas MA (2021). Supercritical CO_2_ extraction of oregano (*Lippia graveolens*) phenolic compounds with antioxidant, α-amylase and α-glucosidase inhibitory capacity. J. Food Meas. Charact..

[4] Petrussa E (2013). Plant flavonoids—Biosynthesis, transport and involvement in stress responses. Int. J. Mol. Sci..

[5] Angst E (2013). The flavonoid quercetin inhibits pancreatic cancer growth in vitro and in vivo. Pancreas.

[6] Lee D (2017). Protective effect of cirsimaritin against streptozotocin-induced apoptosis in pancreatic beta cells. J. Pharm. Pharmacol..

[7] Picos-Salas MA (2022). Naringenin as a natural agent against oxidative stress and inflammation, and its bioavailability. Food Rev. Int..

[8] Rajappa R (2019). Treatment with naringenin elevates the activity of transcription factor Nrf2 to protect pancreatic β-cells from streptozotocin-induced diabetes in vitro and in vivo. Front. Pharmacol..

[9] Frías-Zepeda M, Rosales-Castro M, Escalona-Cardoso G, Paniagua N (2022). Ethanolic extract of *Lippia graveolens* stem reduce biochemical markers in a Murine Model with Metabolic Syndrome. Saudi J. Biol. Sci..

[10] FDA. Q3C—Tables and List Guidance for Industry. https://www.fda.gov/regulatory-information/search-fda-guidance-documents/q3c-tables-and-list-rev-4 (2018).

[11] Lefebvre T, Destandau E, Lesellier E (2021). Selective extraction of bioactive compounds from plants using recent extraction techniques: A review. J. Chromatogr. A.

[12] Chemat F (2019). Green extraction of natural products. Origins, current status, and future challenges. TrAC rends Anal. Chem..

[13] Khaw K-Y, Parat M-O, Shaw PN, Falconer JR (2017). Solvent supercritical fluid technologies to extract bioactive compounds from natural sources: A review. Molecules.

[14] Temelli, F., Saldaña, M. D. A. & Comin, L. Application of supercritical fluid extraction in food processing. in *Comprehensive Sampling and Sample Preparation* (ed. J. Pawliszyn) 415–440 (Academic Press, 2012).

[15] Tyśkiewicz K, Konkol M, Rój E (2018). The application of supercritical fluid extraction in phenolic compounds isolation from natural plant materials. Molecules.

[16] Bimakr M (2011). Comparison of different extraction methods for the extraction of major bioactive flavonoid compounds from spearmint (*Mentha spicata* L.) leaves. Food Bioprod. Process..

[17] Santos SAO, Villaverde JJ, Silva CM, Neto CP, Silvestre AJD (2012). Supercritical fluid extraction of phenolic compounds from *Eucalyptus globulus* Labill bark. J. Supercrit. Fluid..

[18] Kus P, Jerkovic I, Jakovljevic M, Jokic S (2018). Extraction of bioactive phenolics from black poplar (*Populus nigra* L.) buds by supercritical CO_2_ and its optimization by response surface methodology. J. Pharm. Biomed. Anal..

[19] Cardoso C, Afonso C, Lourenço H, Costa S, Nunes ML (2015). Bioaccessibility assessment methodologies and their consequences for the risk–benefit evaluation of food. Trends Food Sci. Technol..

[20] Chen G-L (2015). Total phenolic, flavonoid and antioxidant activity of 23 edible flowers subjected to in vitro digestion. J. Funct. Foods.

[21] Gutiérrez-Grijalva EP, Antunes-Ricardo M, Acosta-Estrada BA, Gutiérrez-Uribe JA, Heredia JB (2019). Cellular antioxidant activity and in vitro inhibition of α-glucosidase, α-amylase and pancreatic lipase of oregano polyphenols under simulated gastrointestinal digestion. Food Res. Int..

[22] Derringer G, Suich R (1980). Simultaneous optimization of several response variables. J. Qual. Technol..

[23] Núñez GA, del Valle JM, de la Fuente JC (2010). Solubilities in supercritical carbon dioxide of (2E,6E)-3,7,11-trimethyldodeca-2,6,10-trien-1-ol (farnesol) and (2S)-5,7-dihydroxy-2-(4-hydroxyphenyl)chroman-4-one (naringenin). J. Chem. Eng. Data.

[24] Al-Rawi SS (2011). The effect of supercritical fluid extraction parameters on the nutmeg oil extraction and its cytotoxic and antiangiogenic properties. Procedia Food Sci..

[25] Atwi-Ghaddar S, Destandau E, Lesellier E (2023). Optimization of supercritical fluid extraction of polar flavonoids from *Robinia pseudoacacia* L. heartwood. J. Utilization..

[26] Bimakr M (2012). Optimization of supercritical carbon dioxide extraction of bioactive flavonoid compounds from spearmint (*Mentha spicata* L.) leaves by using response surface methodology. Food Bioprocess Tech..

[27] Bitencourt RG, Palma AM, Coutinho JAP, Cabral FA, Meirelles AJA (2018). Solubility of caffeic acid in CO_2_+ ethanol: Experimental and predicted data using Cubic Plus Association Equation of State. J. Supercrit. Fluid..

[28] Michielin EMZ (2009). Chemical composition and antibacterial activity of *Cordia verbenacea* extracts obtained by different methods. Bioresour. Technol..

[29] Putra NR (2022). Extraction rate of valuable compounds from peanut skin waste by ethanol-assisted supercritical carbon dioxide: modelling and optimization. Malay. J. Fundamental Appl. Sci..

[30] Liza MS (2010). Supercritical carbon dioxide extraction of bioactive flavonoid from *Strobilanthes crispus* (Pecah Kaca). Food Bioprod. Process..

[31] Xie L (2022). Comparison of flavonoid O-glycoside, C-glycoside and Their Aglycones On Antioxidant Capacity And Metabolism During In Vitro Digestion And In Vivo. Foods.

[32] Bernal-Millán MDJ (2023). Green extracts and UPLC-TQS-MS/MS profiling of flavonoids from Mexican Oregano (*Lippia graveolens*) using natural deep eutectic solvents/ultrasound-assisted and supercritical fluids. Plants.

[33] Morzel M, Canon F, Guyot S (2022). Interactions between salivary proteins and dietary polyphenols: Potential consequences on gastrointestinal digestive events. J. Agric. Food Chem..

[34] Friedman M, Jürgens HS (2000). Effect of pH on the stability of plant phenolic compounds. J. Agric. Food Chem..

[35] Lamothe S, Azimy N, Bazinet L, Couillard C, Britten M (2014). Interaction of green tea polyphenols with dairy matrices in a simulated gastrointestinal environment. Food Funct..

[36] He Q, Lv Y, Yao K (2006). Effects of tea polyphenols on the activities of α-amylase, pepsin, trypsin and lipase. Food Chem..

[37] Gutiérrez-Grijalva EP, Angulo-Escalante MA, León-Félix J, Heredia JB (2017). Effect of in vitro digestion on the total antioxidant capacity and phenolic content of 3 species of Oregano (*Hedeoma patens*, *Lippia graveolens*, *Lippia palmeri*). J. Food Sci..

[38] Junior MRM, Leite AV, Dragano NRV (2010). Supercritical fluid extraction and stabilization of phenolic compounds from natural sources—Review (supercritical extraction and stabilization of phenolic compounds). Open Chem. Eng. J..

[39] Pereira P, Cebola M-J, Oliveira MC, Bernardo-Gil MG (2016). Supercritical fluid extraction vs conventional extraction of myrtle leaves and berries: Comparison of antioxidant activity and identification of bioactive compounds. J. Supercrit. Fluid..

[40] Martínez-Rocha A, Puga R, Hernández-Sandoval L, Loarca-Piña G, Mendoza S (2008). Antioxidant and antimutagenic activities of Mexican Oregano (*Lippia graveolens* Kunth). Plant Foods Hum. Nutr..

[41] Uwineza PA, Gramza-Michałowska A, Bryła M, Waśkiewicz A (2021). Antioxidant activity and bioactive compounds of lamium album flower extracts obtained by supercritical fluid extraction. Appl. Sci..

[42] Odriozola-Serrano I (2023). Stability and bioaccessibility of phenolic compounds in rosehip extracts during in vitro digestion. Antioxidants.

[43] Tagliazucchi D, Verzelloni E, Bertolini D, Conte A (2010). In vitro bio-accessibility and antioxidant activity of grape polyphenols. Food Chem..

[44] Munteanu IG, Apetrei C (2021). Analytical methods used in determining antioxidant activity: A review. Int. J. Mol. Sci..

[45] Galano A (2016). Food antioxidants: Chemical insights at the molecular level. Annu. Rev. Food Sci. Technol..

[46] Ohkatsu Y, Sakurai T, Sato T (2010). Relationship between chemical structure and antioxidant function of flavonoids. J Jpn. Petrol. Inst..

[47] Baranowska M (2021). Interactions between polyphenolic antioxidants quercetin and naringenin dictate the distinctive redox-related chemical and biological behaviour of their mixtures. Sci. Rep..

[48] Qin X, Lu Y, Peng Z, Fan S, Yao Y (2018). Systematic chemical analysis approach reveals superior antioxidant capacity via the synergistic effect of flavonoid compounds in red vegetative tissues. Front. Chem..

[49] Espinosa-Pardo FA, Nakajima VM, Macedo GA, Macedo JA, Martínez J (2017). Extraction of phenolic compounds from dry and fermented orange pomace using supercritical CO_2_ and cosolvents. Food Bioprod. Process..

[50] Quispe-Fuentes I, Uribe E, López J, Contreras D, Poblete J (2022). A study of dried mandarin (*Clementina orogrande*) peel applying supercritical carbon dioxide using co-solvent: Influence on oil extraction, phenolic compounds, and antioxidant activity. J. Food Process. Preserv..

[51] Ferro DM, Mazzutti S, Vitali L, Müller CMO, Ferreira SRS (2019). Integrated extraction approach to increase the recovery of antioxidant compounds from *Sida rhombifolia* leaves. J. Supercrit. Fluid..

[52] Valadez-Carmona L, Ortiz-Moreno A, Ceballos-Reyes G, Mendiola JA, Ibáñez E (2018). Valorization of cacao pod husk through supercritical fluid extraction of phenolic compounds. J. Supercrit. Fluid..

[53] Brodkorb A (2019). INFOGEST static in vitro simulation of gastrointestinal food digestion. Nat. Protoc..

[54] Bernal-Millán MDJ (2022). Spray-dried microencapsulation of Oregano (*Lippia graveolens*) polyphenols with maltodextrin enhances their stability during in vitro digestion. J. Chem.-NY.

[55] Swain T, Hillis WE (1959). The phenolic constituents of *Prunus domestica*. I.—The quantitative analysis of phenolic constituents. J. Sci. Food Agric..

[56] Huang D, Ou B, Hampsch-Woodill M, Flanagan JA, Prior RL (2002). High-throughput assay of oxygen radical absorbance capacity (ORAC) using a multichannel liquid handling system coupled with a microplate fluorescence reader in 96-well format. J. Agric. Food Chem..

[57] Thaipong K, Boonprakob U, Crosby K, Cisneros-Zevallos L, Hawkins Byrne D (2006). Comparison of ABTS, DPPH, FRAP, and ORAC assays for estimating antioxidant activity from guava fruit extracts. J. Food Compost. Anal..

[58] PubChem. https://pubchem.ncbi.nlm.nih.gov/ (2023).

[59] Chemicalize. https://chemicalize.com/app/calculation (2023).

