# Supplementary material for: Supercritical CO2 extraction of naringenin from Mexican oregano (Lippia graveolens): its antioxidant capacity under simulated gastrointestinal digestion
Source: Sci Rep. 2024 Jan 11;14:1146. doi: 10.1038/s41598-023-50997-2 (PMC10784293; doi:10.1038/s41598-023-50997-2)
Supplement: Supplementary file 1 — Supplementary Figure S1. [file 41598_2023_50997_MOESM1_ESM.docx]

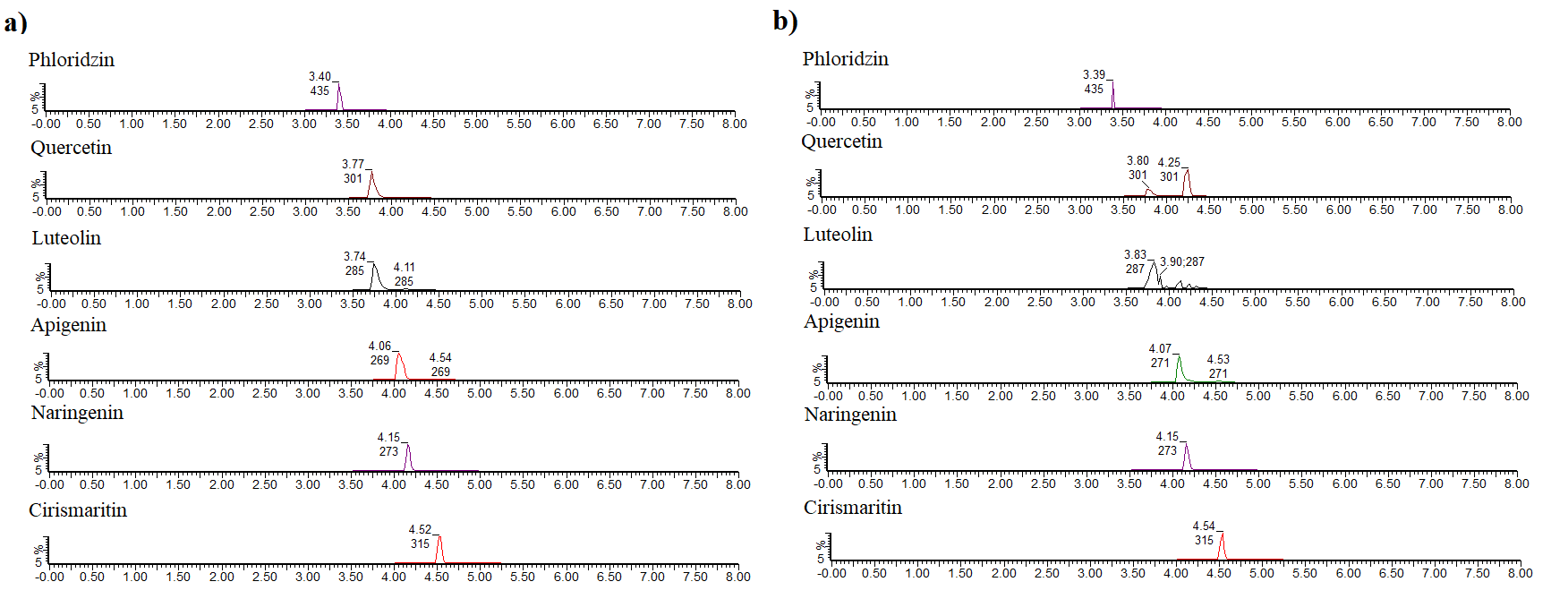


**Supplementary Figure S1**. UPLC-MS chromatograms for the flavonoid standards (a) and the optimized supercritical extract (b).
