# Supplementary material for: Supercritical CO2 extraction of naringenin from Mexican oregano (Lippia graveolens): its antioxidant capacity under simulated gastrointestinal digestion
Source: Sci Rep. 2024 Jan 11;14:1146. doi: 10.1038/s41598-023-50997-2 (PMC10784293; doi:10.1038/s41598-023-50997-2)
Supplement: Supplementary file 2 — Supplementary Figure S2. [file 41598_2023_50997_MOESM2_ESM.docx]

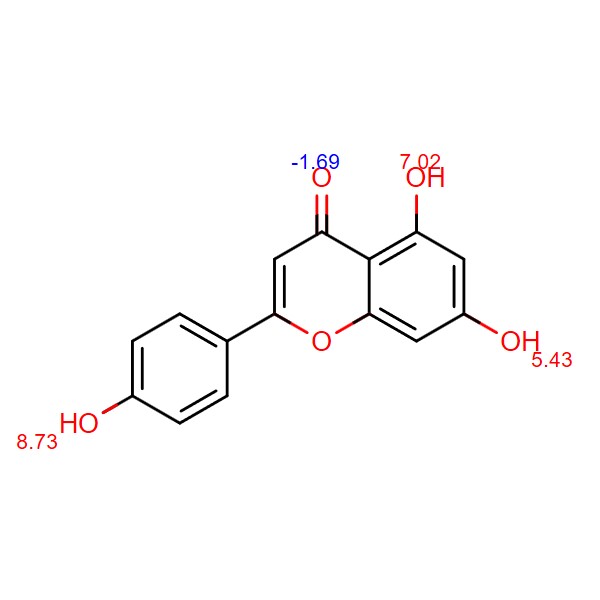

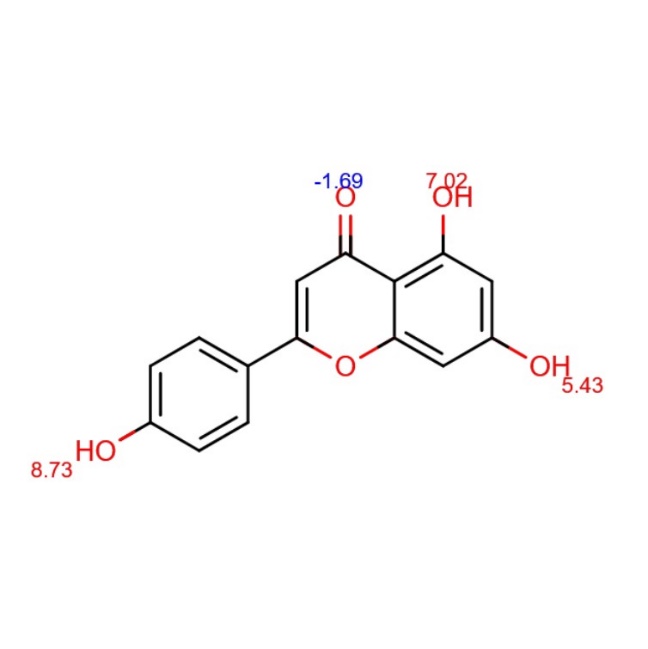


b)

a)


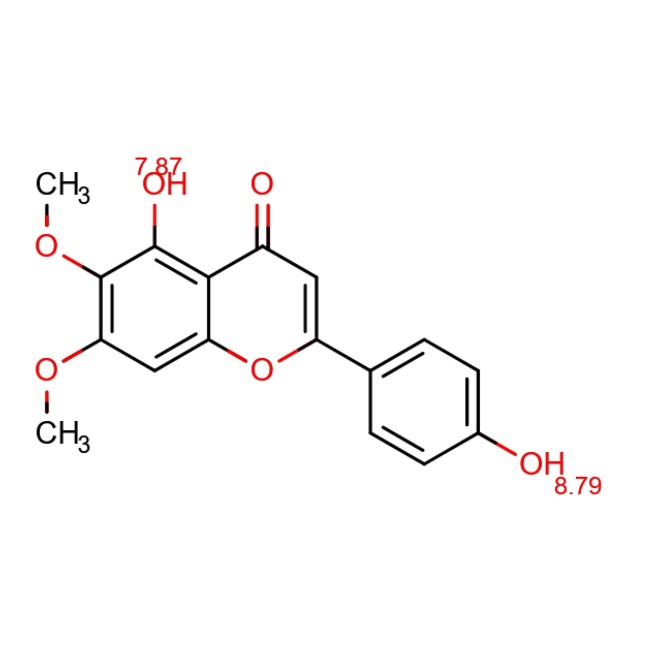

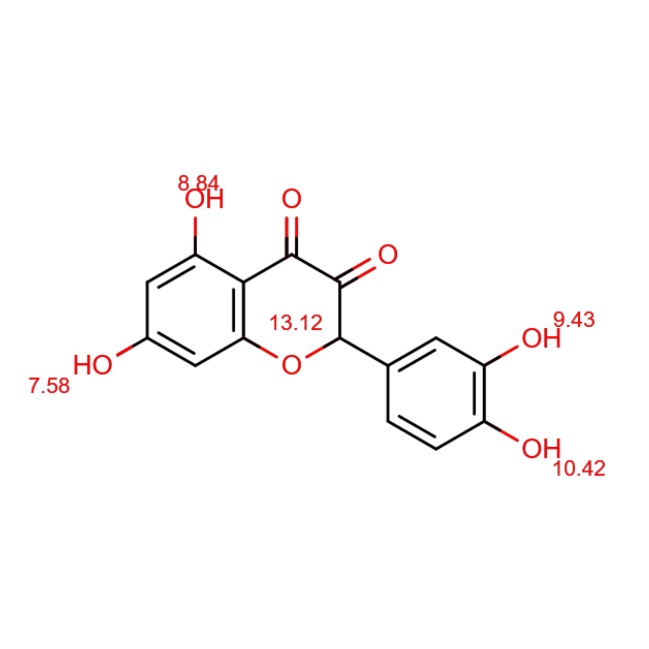


c)

c)

**Supplementary Figure S2**. pKa structures of a) Apigenin, b) naringenin, c) cirsimaritin, d) quercetin.
