# Supplementary figures and images for: Supercritical CO2 extraction of naringenin from Mexican oregano (Lippia graveolens): its antioxidant capacity under simulated gastrointestinal digestion
Source: Sci Rep. 2024 Jan 11;14:1146. doi: 10.1038/s41598-023-50997-2 (PMC10784293; doi:10.1038/s41598-023-50997-2)

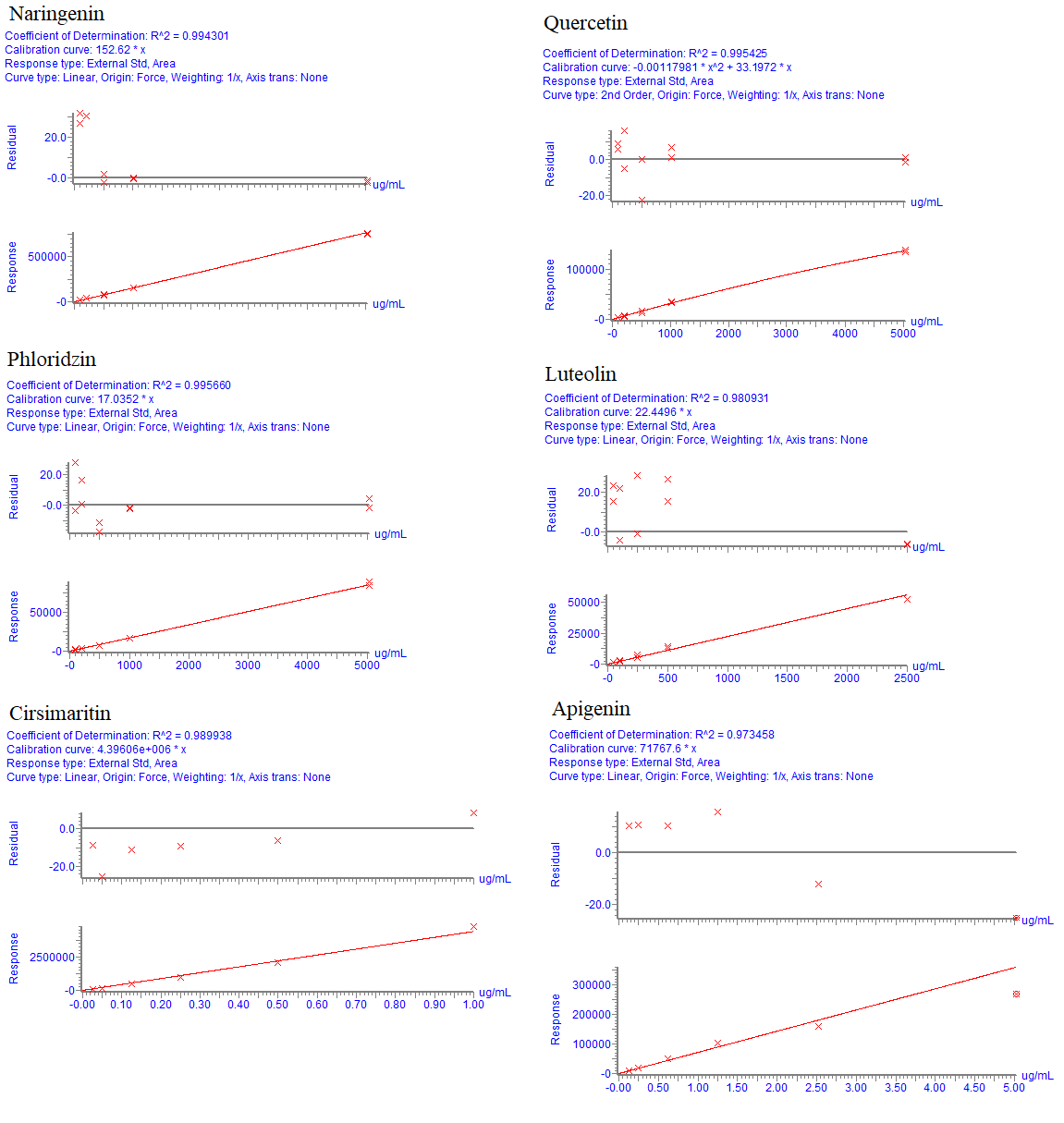


**Supplementary Figure S3**. Calibration curves of flavonoid standards.

Supplement: Supplementary file 3 — Supplementary Figure S3. [file 41598_2023_50997_MOESM3_ESM.docx]
