# Supplementary material for: Supercritical CO2 extraction of naringenin from Mexican oregano (Lippia graveolens): its antioxidant capacity under simulated gastrointestinal digestion
Source: Sci Rep. 2024 Jan 11;14:1146. doi: 10.1038/s41598-023-50997-2 (PMC10784293; doi:10.1038/s41598-023-50997-2)
Supplement: Supplementary file 4 — Supplementary Table S1. [file 41598_2023_50997_MOESM4_ESM.docx]

| **Salt – Concentration** | **Oral solution (mL)**  **pH 7.0** | **Gastric solution (mL)**  **pH 3.0** | **Intestinal solution (mL)**  **pH 7.0** |
| --- | --- | --- | --- |
| KCl – 37 g/L | 1.8875 | 0.8625 | 1.7 |
| KH_2_PO_4_ – 68 g/L | 0.4625 | 0.1125 | 0.2 |
| NaHCO_3_ – 84 g/L | 0.85 | 1.5625 | 10.625 |
| NaCl – 117 g/L | - | 1.475 | 2.4 |
| MgCl_2_ – 30.5 g/L | 0.0625 | 0.05 | 0.275 |
| (NH_4_)_2_CO_3_ – 48 g/L | 0.0075 | 0.0625 | - |
| HCl – 6 M | 0.01125 | 0.1625 | 0.175 |

**Supplementary Table S1**. Volumes of electrolytes stock solution for a volume of 50 mL (oral and gastric) and 100 mL (intestinal) diluted with distilled water.
